# Health Impact Assessment of a Predicted Air Quality Change by Moving Traffic from an Urban Ring Road into a Tunnel. The Case of Antwerp, Belgium

**DOI:** 10.1371/journal.pone.0154052

**Published:** 2016-05-11

**Authors:** Daan Van Brusselen, Wouter Arrazola de Oñate, Bino Maiheu, Stijn Vranckx, Wouter Lefebvre, Stijn Janssen, Tim S Nawrot, Ben Nemery, Dirk Avonts

**Affiliations:** 1 Department of Family Medicine and Primary Health Care, Ghent University, Ghent, Belgium; 2 Director of VRGT (Flemish Society of Respiratory Health), Brussels, Belgium; 3 VITO, Flemish Institute of Technologic Research, Mol, Belgium; 4 Centre for Environmental Sciences, Hasselt University, Hasselt, Belgium; 5 Department of Public Health & Primary Care, University of Leuven, Leuven, Belgium; Beihang University, CHINA

## Abstract

**Background:**

The Antwerp ring road has a traffic density of 300,000 vehicles per day and borders the city center. The ‘Ringland project’ aims to change the current ‘open air ring road’ into a ‘filtered tunneled ring road’, putting the entire urban ring road into a tunnel and thus filtering air pollution. We conducted a health impact assessment (HIA) to quantify the possible benefit of a ‘filtered tunneled ring road’, as compared to the ‘open air ring road’ scenario, on air quality and its long-term health effects.

**Materials and Methods:**

We modeled the change in annual ambient PM_2.5_ and NO_2_ concentrations by covering 15 kilometers of the Antwerp ring road in high resolution grids using the RIO-IFDM street canyon model. The exposure-response coefficients used were derived from a literature review: all-cause mortality, life expectancy, cardiopulmonary diseases and childhood Forced Vital Capacity development (FVC).

**Results:**

Our model predicts changes between -1.5 and +2 μg/m³ in PM_2.5_ within a 1,500 meter radius around the ring road, for the ‘filtered tunneled ring road’ scenario as compared to an ‘open air ring road’. These estimated annual changes were plotted against the population exposed to these differences. The calculated change of PM_2.5_ is associated with an expected annual decrease of 21 deaths (95% CI 7 to 41). This corresponds with 11.5 deaths avoided per 100,000 inhabitants (95% CI 3.9–23) in the first 500 meters around the ring road every year. Of 356 schools in a 1,500 meter perimeter around the ring road changes between -10 NO_2_ and + 0.17 μg/m³ were found, corresponding to FVC improvement of between 3 and 64ml among school-age children. The predicted decline in lung cancer mortality and incidence of acute myocardial infarction were both only 0.1 per 100,000 inhabitants or less.

**Conclusion:**

The expected change in PM_2,5_ and NO_2_ by covering the entire urban ring road in Antwerp is associated with considerable health gains for the approximate 352,000 inhabitants living in a 1,500 meter perimeter around the current open air ring road.

## Background

The Global Disease Burden Study ranks exposure to indoor and outdoor air pollution as the third and ninth leading risk factors for all-cause mortality worldwide, respectively, with indoor air pollution responsible for about 3.5 million deaths and outdoor air pollution for 3.2 million deaths each year [1].

It is known that long-term residential proximity to major roadways is associated with an increased risk of all-cause mortality, as well as with asthma, diminished lung function, adverse birth outcomes and childhood cancer [2, 3]. Exposure to road traffic is the largest population attributable risk factor for triggering acute myocardial infarction [4]. A recent observational study in Vancouver showed that moving away from a major roadway was associated with a decreased risk of cardiovascular mortality, suggesting that interventions to avoid traffic exposure may be beneficial [5]. The groups most susceptible to adverse health effects of air pollution are children, adults over 65 years of age, persons with chronic diseases such as asthma, chronic obstructive pulmonary disease, cardiac ischemia, and pregnant women due to prenatal exposure of the fetus [6, 7].

The negative health effects of short-term exposure to air pollution are well recognized, however several epidemiological data suggest that long-term exposure may be more important in terms of overall public health impact [8, 9].

### Air quality in Belgium and Antwerp

The Flemish Environmental Agency (VMM) showed that traffic in Belgium is currently a bigger environmental threat than the industrial sector [10]. Belgium is located in one of the most densely populated regions in Western Europe. In Flanders, environmental pollution causes 8 percent of the total disease burden, 75 percent of this pollution is attributed to particulate matter. [10] The European Commission’s Clean Air for Europe (CAFE) study indicates that for Belgian citizens the loss of life expectancy attributable to the exposure of anthropogenic PM_2.5_ is on average 13.2 months, as compared to the European average of 8.1 months, making it the worst performer of all European countries [11]. Studies on health economics, by the European Environment Agency among others, show that direct and indirect health costs of air pollution in Belgium may be as high as 5 billion euros a year. The main polluter is observed as traffic and transportation [10].

The Antwerp ring road concentrates most of the traffic in the metropolitan region. Every day 300,000 vehicles (of which 27% are freight vehicles = 81,000) use the most populated road link (half of it in one direction and the other half in the other direction); 25% of it is ongoing traffic (+- 75,000 cars per day) [12]. The historical location of the Antwerp ring road—cutting through areas with population densities reaching above 11,000 inhabitants/km² (e.g. in district ‘Borgerhout’)–is thought to increase the negative effects of air pollution on health. More or less 352,000 people live within a 1,500m radius from the ring road. Within a 500m perimeter 55 schools, hospitals and geriatric homes are situated. 12 schools and 75 nurseries are located in the area where daily average particulate concentrations were calculated to exceed European limits [13].

**Fig 1:**
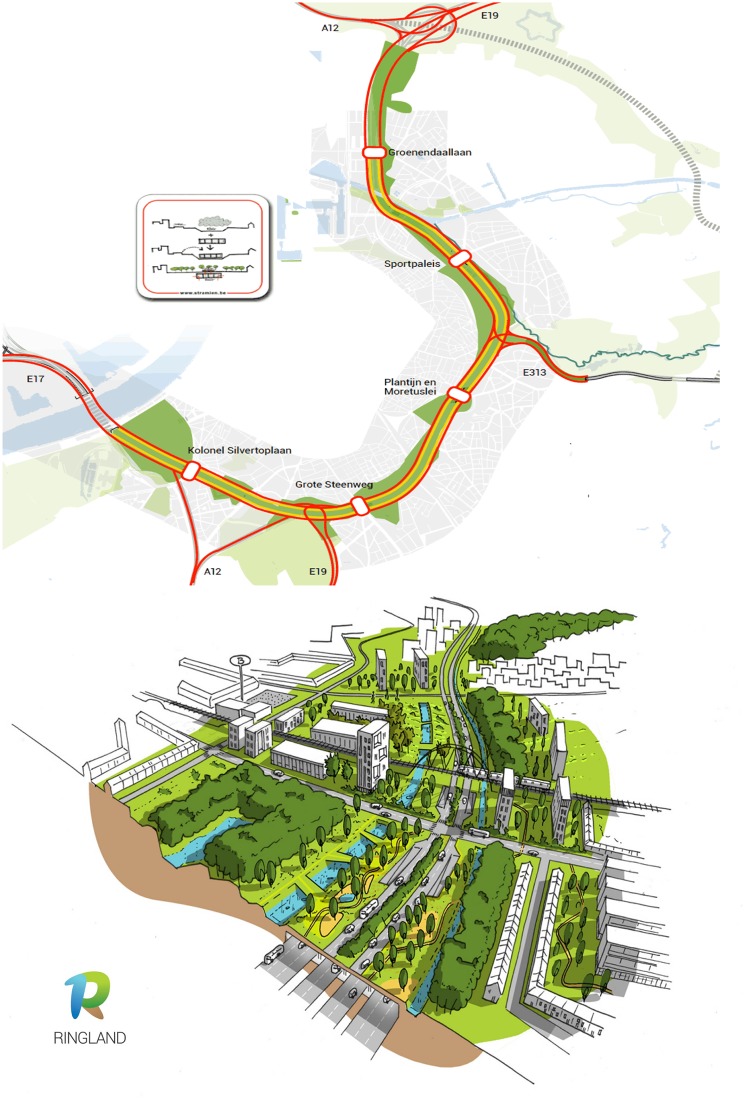
Overview and visual representation of the ‘Ringland’ project (www.ringland.be). Reprinted from ‘Ringland’ under a CC BY license, with permission from Peter Vermeulen, original copyright 2015.

In Antwerp, the official plans for expansion and extension of the existing ring road have initiated widespread and sustained public debate over the last 10 years. This debate has raised awareness about the health impact of air and noise pollution and resulted in community driven development of alternative solutions. Citizen groups have proposed an alternative trajectory leading traffic further away from the city center where population densities are lower, and covering the entire urban section of the ring road (15 kilometers long)–with a view to reducing noise and air pollution and of creating open space on top of the ring road, enabling the development of public parks and buildings [14] (Fig 1). In this article we present a health impact assessment (HIA) of the predicted air quality changes of a ‘filtered tunneled ringroad’ scenario (the ‘Ringland project’) as compared to the ‘open air ringroad’.

Despite the fact that only 10% of PM_2.5_ emissions are traffic-related in Antwerp [15], we focus on particulate matter with an aerodynamic diameter of less than 2.5 μg/m^3^ in our health impact assessment because the existing associations between long-term exposure to PM_2.5_ and mortality/morbidity are the strongest described in medical literature. [9].

We also assess the impact of the predicted NO_2_ reduction on health in our HIA, since this parameter has been strongly associated with differences in lung function and pulmonary health outcomes [16]. In the urban area of Antwerp 44% of NO_2_ in outdoor air is traffic-related [15].

## Materials and Methods

### Air pollution reduction scenario: Ringland project

In this study we analyse the change in annual concentrations of PM_2.5_ and NO_2_ and assess the health impact, predicted by the ‘Ringland project’—moving traffic from the entire urban ring road (15 kilometers long) into a tunnel and filtering the air pollution. We examined and compared a ‘filtered tunneled ring road’ (the ‘Ringland project’), as compared to an ‘open air ring road’.

Differences in annual PM_2.5_ and NO_2_ concentrations were calculated using the RIO—Immision Frequency Distribution (IFDM) street canyon model OSPM (Operational Street Pollution Model) [17]. RIO is a geospatial interpolation model which provides urban background concentrations at a resolution of 4x4 km^2^ based upon the Belgian Air quality monitoring network. The detailed effect of traffic and point sources are calculated using the IFDM Gaussian Dispersion model, using an irregular grid with a total of 143,416 receptor points and highest resolution near the roads close to 20 m resolution [18]. The coupling between the background model and the high-resolution dispersion model, its validity and performance have been well-documented in the international literature [17]. Based on detailed information of Antwerp’s building configuration, the city’s street canyons are identified. These urban roads confined by continuous building-walls have increased pollutant concentrations as ventilation is reduced. Here IFDM’s street canyon module is applied which is a simplification of the OSPM-module [19, 20].

Emissions were modeled by MIMOSA v4.25 (the official Flemish traffic emission model), taking into account changes in traffic situation and fleet composition predicted by 2020 [21, 22]. This emission model is based on the COPERT methodology. [23] The future traffic intensities and vehicle speeds are calculated in a mobility study by the Flemish Traffic Center and have been adapted to the Ringland scenario by a mobility consultancy office, Vectris. The calculation of emissions starts from daily traffic volumes. Relying on the COPERT methodology, emission factors are applied to generate hourly output for the different pollutants, NO_2_, NO_X_ and PM_2.5_. [24] PM emissions include exhaust and non-exhaust emissions. [24] The ‘Ringland project’ turns the ring road into a tunnel complex and moves the ‘Singel’—the inner ring road which is not a highway—to a new location on top of the tunnels. The main effect a tunnel has on the air quality is a re-distribution of the traffic emissions along the tunnel trajectory to the tunnel portals. These traffic emissions were redistributed in this study using an analytical model based upon the Hardy-Cross method [25, 26]. In this method the tunnels are represented by a network of segments and nodes. At each node conservation of mass is imposed and for each segment, conservation of energy. Along each segment pressure “losses” are taken into account due to wall friction and the vehicle piston effect [27]. This leads to a system of nonlinear equations which are solved iteratively using MATLAB. From this model, the average flowrates at each of the exits were calculated depending on the distribution, composition, speed and intensities of the traffic in the different segments and the tunnel geometry. More details about this method are explained in the supplementary material. (S1 File Supplementary Modeling Information) Next to a scenario without pollutant filtering, a hypothetical scenario was calculated in which filter installations would be placed at exits and junctions. It was suggested, based on field trials, that a filtering efficiency of 65% [28] can be obtained for the fraction of the air flow which passes through the installation (typically 30%) [29].

### Identification of population at risk and selection of health outcomes

Based on the evidence that traffic-related health effects can be measured up to 1,500 meters from a major road, we investigated reductions in PM_2.5_ and NO_2_ in radiuses in the first 1,500 meters around the ring road [16]. The exposure to PM_2,5_ and NO_2_ and the reduction of this exposure has been analyzed by the linking of the detailed population map of Antwerp with the air quality results. Population data are available in high resolution at address location and exposure has been calculated on this high resolution, with results aggregated in radiuses around the Ring Road to respect privacy.

Clinically relevant health outcomes were selected from the international medical literature, using a PubMed search for ‘Improved Air Quality’ (limited to title and abstract), supplemented by more epidemiological studies on the health impact of air pollution referred to in these studies. In order to select studies on which to base our HIA of the predicted PM_2.5_ and NO_2_ reduction, we considered a number of factors: the studies must focus on long term differences in mortality or cardiovascular/pulmonary outcomes, they must be peer-reviewed, they must account for potential confounders, they must be recent—using advanced analytical methods -, and cover longer periods and larger populations.

We identified 32 articles on health outcomes of PM_2.5_ and NO_2_ reduction that would be appropriate for evaluation in our study. For an extensive description of our scope review of these articles, please refer to the addendum. (S7 File Addendum) In the two largest prospective cohort studies based in the United States, the American Cancer Society (ACS) Cancer Prevention Study, and the Harvard Six Cities Study (SCS), robust associations were demonstrated between long-term exposure to concentrations of ambient air pollution and mortality from cardiopulmonary diseases, after adjusting for smoking and other risk factors. The SCS also has an extended follow-up study, describing what happened after air quality improvement [9, 30–32]. The Environmental Protection Agency (EPA) has conducted benefit assessments for PM_2.5_ reduction and these assessments have also undergone peer review of the analytical approaches used, including the choice of Concentration Response Function. The conclusion is generally that ACS and SCS remain the preferred basis to estimate mortality [33, 34]. Only the SCS follow-up study extensively describes an all-cause mortality improvement after air quality improvement [32]. A variety of studies has now also documented the relationship between decreased exposure to air pollution (partly because of measures taken by governments) and decreases in population mortality and morbidity as well as increases in life expectancy [35, 36].

Because it is known that vulnerable populations are significantly more affected by air pollution, we have chosen to specifically take into account the health impact on children going to school in the 1,500 meter radius of the ring road, in which effects are known to be present [6, 7, 16, 37, 38]. Furthermore the number of elderly people in nursing homes in the 1,500 meter radius around the Ring Road was counted, because a history of frailty is known to amplify the adverse health outcomes [39].

### Calculation of health impact

The Concentration Response Functions used in our study correspond to the relative risks (RR)–or a pooled RR—found in prospective air quality improvement studies that show a relevant health gain associated with a decrease in PM_2.5_ and NO_2_ outdoors. Focusing on studies about the health effects of decreased exposure to air pollution and taking into account the aforementioned inclusion criteria, we identified the *extended follow-up of the Harvard Six Cities Study by Laden et al*. as the most reliable study for the calculation of the changes in mortality (RR of 0.73 (CI 0.57–0.95) per 10 mcg/m^3^ decrease of annual PM_2.5_ concentration); *Pope at al*. as the most reliable for the calculation of life expectancy (+ 0.61y (+- 0.2y) per 10 mcg/m3 decrease of annual PM_2.5_ concentration), and *Gauderman et al*. as the most reliable to predict changes in childhood lung function (FVC difference +168.9 ml (CI 127–210.7) per 14.1 ppb decrease of annual NO_2_ concentration) [32, 36, 38].

However, given the scarcity of studies on air quality improvement, we also used the RR found in two observational epidemiological studies based on air quality change, and not specifically on air quality improvement: two meta-analyses of epidemiological studies based on air pollution were taken to calculate the differences in lung cancer mortality and the incidence of myocardial infarctions [40, 41].

Data on mortality, life expectancy and lung cancer mortality were obtained from the Flemish ‘Care and Health Agency’ [42]. Data on the incidence of myocardial infarction in the region were obtained from the ‘Dutch Family Doctors Association’ [43].

## Results

**Fig 2:**
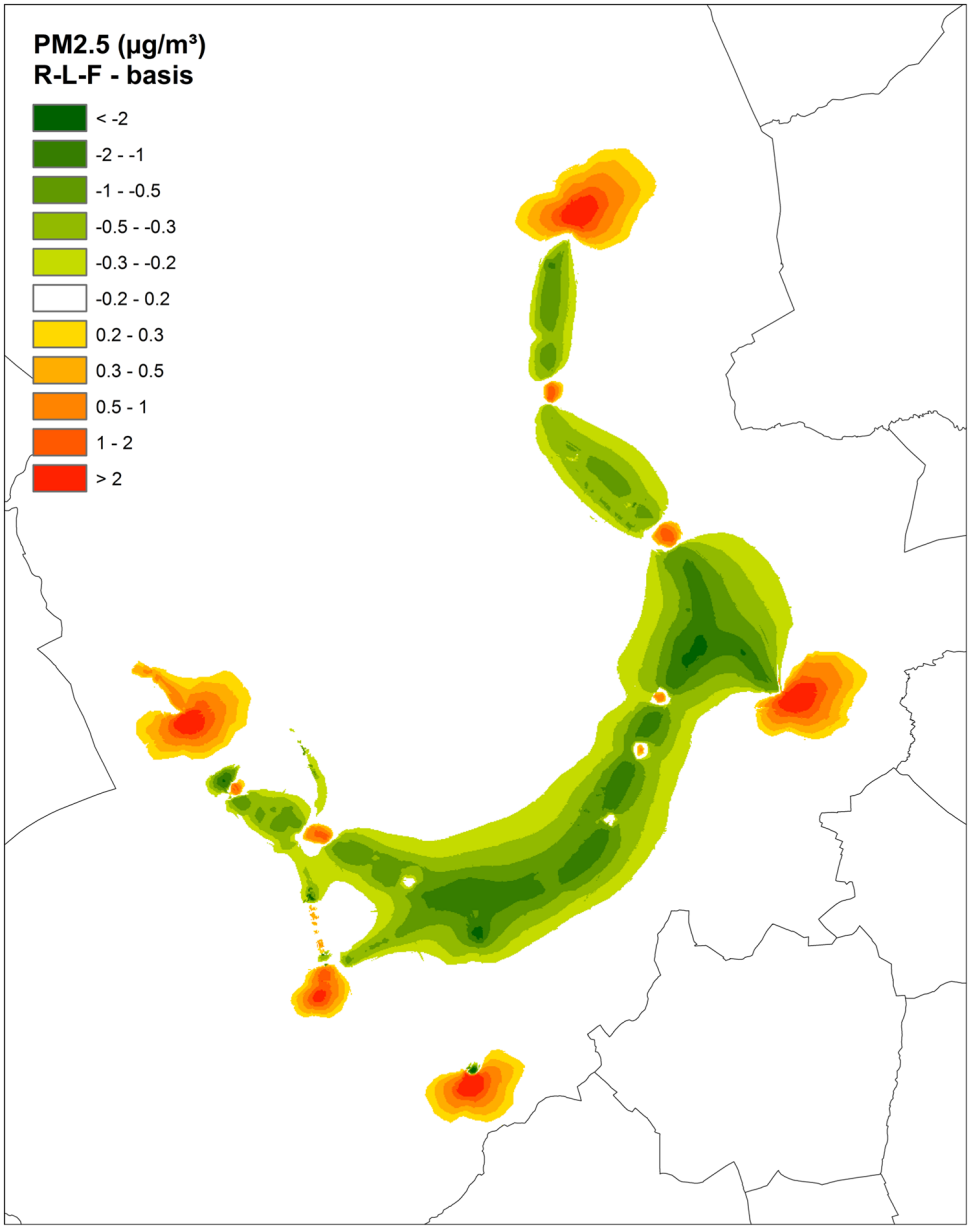
PM_2,5_ difference of scenario RL-F (‘Ringland’ with filtration—a filtered tunneled ring road) with the basic scenario (‘open air ring road’).

**Fig 3:**
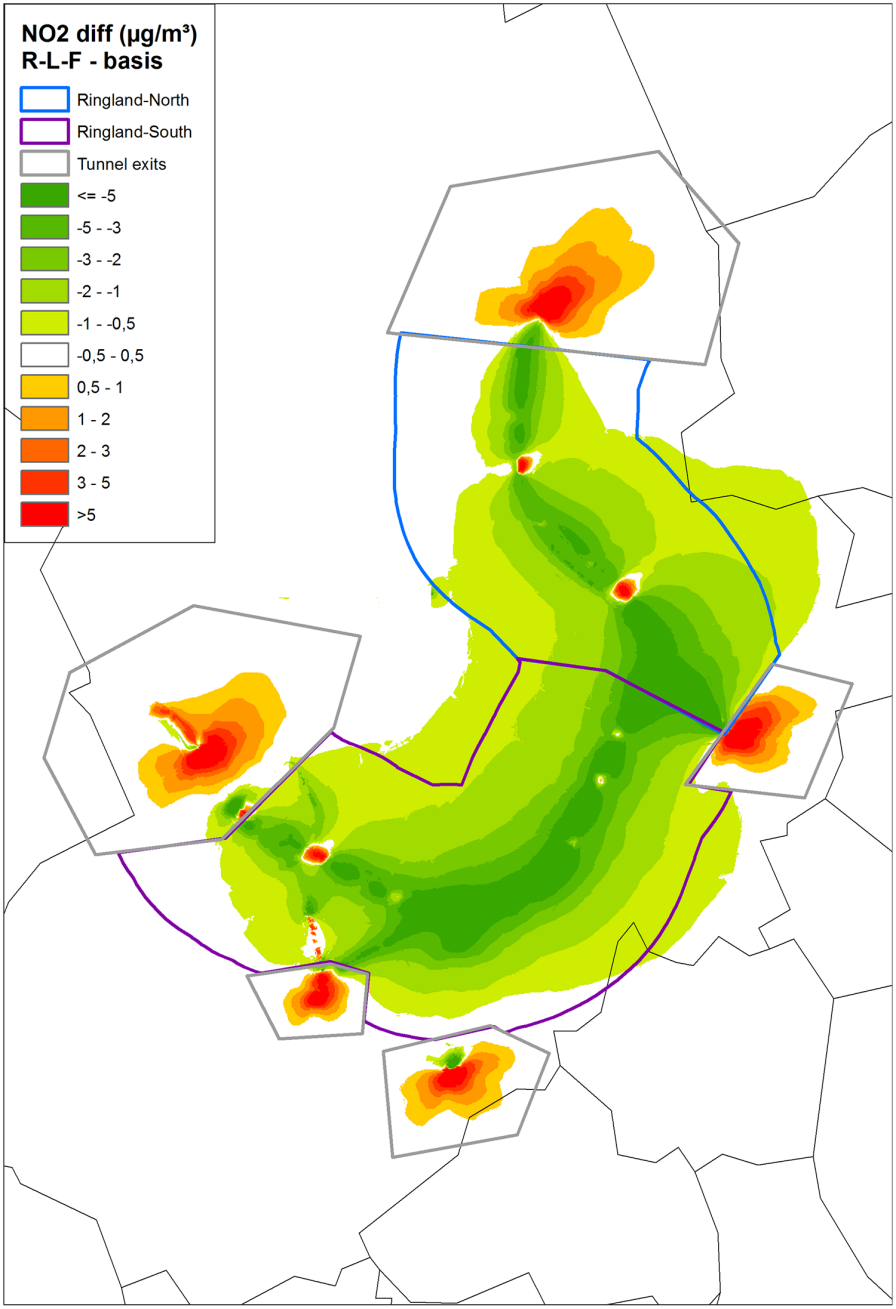
NO_2_ difference of scenario RL-F (‘Ringland’ with filtration—a filtered tunneled ring road) with the basic scenario (‘open air ring road’). In addition the ‘Ringland North’, ‘Ringland South’ and ‘Tunnel Mouth’ areas are shown.

**Fig 4:**
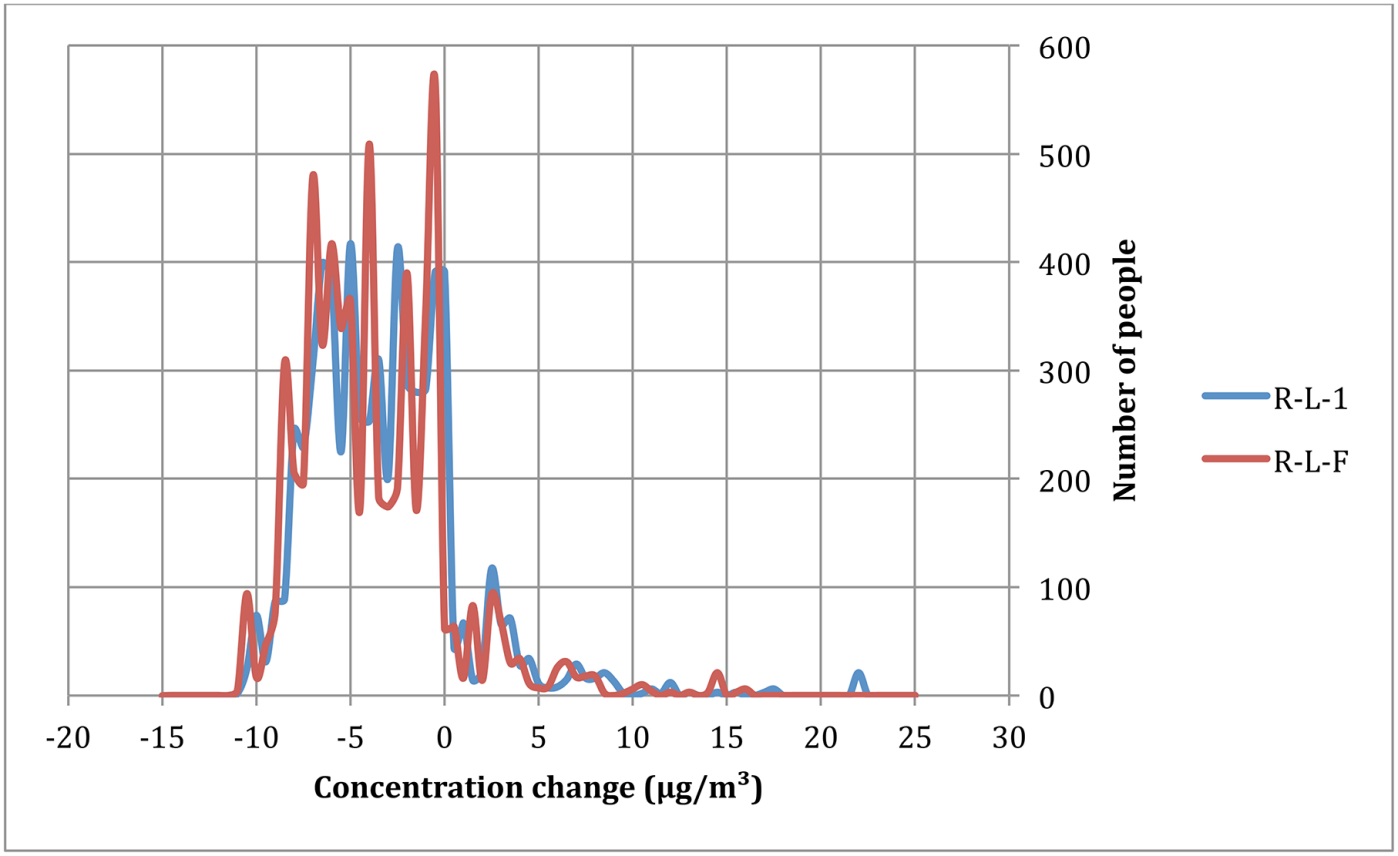
Example of NO_2_ difference of RL-1 (‘Ringland’ without filtration—a tunneled ring road) and RL-F (‘Ringland’ with filtration—a filtered tunneled ring road) with the basic scenario (‘open air ring road’). All are plotted against population exposed to this difference, for the 50-100m radius around urban ring road.

Considerable reductions of annual PM_2.5_ and NO_2_ concentrations were found in the 1,500m radius around the ring road when comparing the ‘Ringland project’ (a filtered tunneled ring road) with an ‘open air ring road’ scenario (Figs 2–4).

Even after filtering, a tunneled ring road displaces a lot of traffic emissions from the ring road close to the city center—where the population density is high—to the tunnel exits of the ‘Ringland’ plan. As a consequence, elevated annual concentration differences of PM_2.5_ and NO_2_ can be seen on the maps near the exits of the tunnel complex and partly also at the motorway exits (Figs 2 and 3).

When the RR of our concentration response function for mortality associated with PM_2.5_ is applied to the population exposed to the PM_2.5_ differences described in Table 1, the estimated reduction of PM_2.5_ in the ‘filtered tunneled ring road’ scenario could prevent overall 21 annual premature deaths (95% CI -7 to -41) in a 1,500 meter perimeter (including the tunnel mouths were the mortality increases) (Tables 1 and 2). This corresponds with 6 deaths avoided per 100,000 inhabitants (95% CI -2 to -12) in the first 1,500 meter around the ring road and 11.5 death avoided per 100,000 inhabitants (95% CI 3.9–23) in the first 500 meters around the ring road every year. An interesting finding was that 8 of the 21 avoided deaths around the ring road were found in the radius between 500 and 1,000 meter around the ring road (Table 2). In the ‘Ringland North’ area—the Northern region of Antwerp (Fig 3) 7.9 premature deaths could be avoided per 100,000 inhabitants (95% CI -2.7 to -15.8), as compared to 10.3 deaths avoided per 100,000 inhabitants (95% CI -3.4 to -20.6) in the ‘Ringland South’ area (Fig 3 and Table 3). At the (less densely populated) tunnel mouths however, the annual mortality increases with 4.7 deaths (95% CI +1.6 to +9.4) (Tables 3 and 4). A total of 1,710 (+-570) overall life years could be gained annually by tunneling the ring road (Table 2).

**Table 1:** Population exposed to PM_2,5_ change—comparing the ‘Ringland with filtration’ scenario (filtered tunneled ring road) with the basic scenario (open air ring road)–in a 500 meter perimeter and a 1,500 meter perimeter around the ring road.

PM_2,5_ delta between ‘filtered tunneled ring road’ and ‘open air ring road’	Population exposed in 500 m radius	Population exposed in 1500 m radius
-2	0	0
-1.75	0	0
-1.5	25	25
-1.25	244	244
-1	1,518	1,518
-0.75	3,416	3,416
-0.5	13,581	13,581
-0.25	46,925	97,613
0	29,603	218,280
0.25	7,041	11,017
0.5	3,219	3,219
0.75	1,253	1,253
1	422	422
1.25	538	538
1.5	290	290
1.75	96	96
2	54	54
TOTAL POPULATION EXPOSED		
	108,225 residents	351,566 residents

**Table 2:** Differences in all-cause mortality, life expectancy, number of myocardial infarctions and lung cancer deaths in the exposed population—predicted by the ‘filtered tunneled ring road’ scenario (‘Ringland project’) as compared to the ‘open air ring road’. (dose response functions based on the *extended follow-up of the Harvard Six Cities Study by Laden et al*. *2006* for the calculation of the changes in mortality [32]; *Pope at al*. *2009* for the calculation of life expectancy [36]; *a meta-analysis by Nawrot et al*. *2014* for the calculation of myocardial infarctions [41]; and a meta-analysis by *Hamra et al*. *2014* for the calculation of lung cancer deaths [40]).

	500m radius of ring road	1500m radius of ring road
Population exposed	108,225	351,556
Annual number of deaths avoided	12.5 (95% CI 4.2–24.9)	21.1 (95% CI 7–41)
Annual number of deaths avoided per 100,000 inhabitants	11.5 (95% CI 3.9–23)	6 (95% CI 2–12)
Annual total number of life years gained	1009.7 (+- 336.6)	1710.4 (+-570.1)
Annual number of myocardial infarctions avoided	0.3 (95% CI 0–0.7)	0.5 (95% CI 0–1.13)
Annual number of lung cancer deaths avoided	0.05 (95% CI 0.02–0.07)	0.1 (95% CI 0.04–0.12)

**Table 3:** Annual all-cause mortality changes comparing the ‘filtered tunneled ringroad’ scenario (‘Ringland project’) with the ‘open air ring road’ in 3 particular areas: ‘Ringland North’, ‘Ringland South’ and ‘Tunnel Mouth’ areas. (dose response function based on the *extended follow-up of the Harvard Six Cities Study by Laden et al*. *2006* [32]).

	Number of inhabitants	Change in annual number of deaths
‘Ringland North’ area	109,043	- 8.6 (95% CI -2.9 to -17.2)
‘Ringland South’ area	169,505	- 17.4 (95% CI -5.8 to -34.9)
Tunnel mouth areas	49,992	+4.7 (95% CI +1.6 to +9.4)

**Table 4:** Population exposed to PM_2,5_ changes near the tunnel mouths predicted by the model, comparing the ‘filtered tunneled ringroad’ with the ‘open air ringroad’ scenario.

PM_2,5_ delta ‘filtered tunneled ringroad’ versus ‘open air ringroad’.	Population exposed
-2	0
-1.75	0
-1.5	0
-1.25	0
-1	0
-0.75	0
-0.5	39
-0.25	203
0	34,384
+0.25	10,224
+0.5	2,633
+0.75	1,155
+1	642
+1.25	277
+1.5	292
+1.75	98
+2	45
TOTAL POPULATION EXPOSED	
	49,992

When comparing the ‘filtered tunneled ring road’ scenario with the ‘open air ring road’ in 356 out of 430 schools in the 1,500 meter radius, the model predicts an improvement of Forced Vital Capacity (FVC) development in school age children, based on the reduction of annual NO_2_ concentrations (Table 5). In 41 schools the predicted Forced Vital Capacity (FVC) development gain in children aged between 11 and 15 was between 15ml and 64ml. In 315 other schools children could still benefit from a smaller FVC gain of 3 to 10ml (Table 5).

**Table 5:** Changes in Forced Vital Capacity (FVC) development in children as predicted by the model, comparing the ‘filtered tunneled ring road’ scenario versus the ‘open air ring road’, based on the predicted annual NO_2_ concentration changes in 430 schools in the 1,500 meter perimeter. (dose response function based on *Gauderman et al*. [38]).

N0_2_ delta ’filtered tunneled ring road’ versus ‘open air ring road’.	Number of schools	Mean FVC gain (ml)	Confidence interval	
-10	1	+64.0	+48.0	+79.0
-7.4	1	+47.4	+35.5	+58.5
-5.14	8	+32.9	+24.6	+40.6
-4.51	7	+28.9	+21.6	+35.6
-3.51	3	+22.4	+16.8	+27.7
-2.44	21	+15.6	+11.7	+19.3
-1.35	98	+8.7	+6.5	+10.7
-0.49	217	+3.2	+2.4	+3.9
0	2	0.0	0,0	0.0
+0.17	72	-1.1	-0.8	-1.3
Total number of schools				
	430			

The estimated decrease in lung cancer mortality and decline in the incidence of acute myocardial infarction between the ‘filtered tunneled ring road’ versus ‘open air ring road’ were borderline relevant: 0,03 (CI 95%—0.01 to 0.3) and 0.1 (CI 95% 0 to 1.13) per 100,000 inhabitants respectively (Table 2).

## Discussion

Considerable overall health gains for the approximately 352,000 inhabitants living in a 1,500m perimeter around the current ring road were estimated for the ‘filtered tunneled ring road’ plan (the ‘Ringland project’). The most important outcomes of our model, comparing a ‘filtered tunneled ring road’ with an ‘open air ring road’, are the predictions of an annual decrease of 21 deaths (95% CI 7 to 41) in the population exposed to a PM_2.5_ reduction. Furthermore 356 out of 430 schools in the 1,500 meter perimeter, experience declining annual NO_2_ concentrations, corresponding with an improvement of Forced Vital Capacity development between 3 and 64ml among children between 11 to 15 years old.

Over the past 20 years, the health impact assessment (HIA) has served as a methodological tool used by decision makers to quantify and evaluate the impact of interventions on air pollution on human health [44, 45]. Premature mortality is the most performant parameter to evaluate the global health impact of the population exposed to air pollution [33].

We focused in our HIA on particulate matter with an aerodynamic diameter of less than 2.5 μg/m^3^ because the existing associations between long-term exposure to PM_2.5_ and mortality/morbidity are the strongest as described in medical literature. [9]. We also chose to assess the impact of the predicted NO_2_ reduction on health in our HIA, since long-term exposure to this parameter has recently been strongly associated with differences in childhood lung function [16, 38].

A variety of studies has now documented the relationship between annual PM_2.5_ declines—partly because of interventions by governments—and decreases in population mortality/morbidity as well as increases in life expectancy [32, 35, 36]. Currently the findings in the ACS and SCS provide the highest grade of evidence that long-term exposure to fine particulate air pollution is a crucial risk factor of all-cause, cardiopulmonary and lung cancer mortality. These associations were observed even after controlling for cigarette smoking, Body Mass Index, diet, occupational exposure, other individual risk factors, and for regional and other spatial differences [9, 32].

The SCS follow-up study is the only large-scale study extensively describing what happened to all-cause mortality after air quality improvement [32]. Because the ‘Ringland project’ specifically focuses on air quality improvement, we have chosen to use the SCS follow-up as a dose response function for all-cause mortality, and not the ACS, because this does not focus on air quality improvement.

The findings in the SCS follow-up study however suggest that mortality effects, described in earlier epidemiological studies, not focused on air quality improvement but on air pollution in general, may be at least partially reversible [32]. Therefore it is reasonable to assume that improved air quality could (at least partially) minimize other detrimental health outcomes associated with traffic related air pollution. That is why we have also used the RR found in two observational epidemiological studies, based on air quality change and not specifically on air quality improvement, for three ‘smaller’ outcomes: we namely used two meta-analyses of epidemiological studies about air pollution to calculate the differences in lung cancer mortality and the incidence of myocardial infarctions [40, 41].

**Fig 5:**
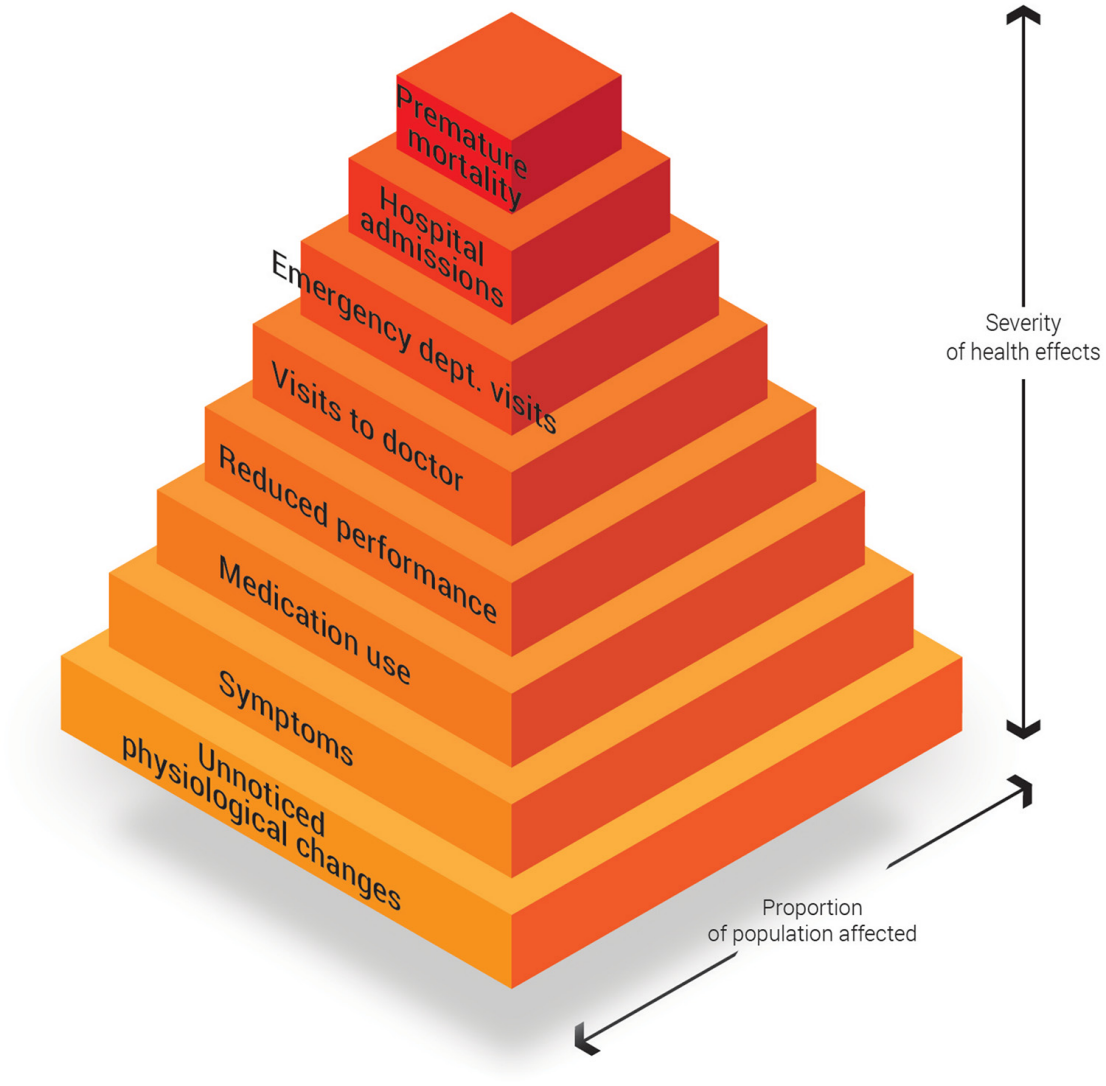
The ‘pyramid of health’ effects (by Künzli et al. [46]) associated with air pollution’. This pyramid shows that the number of people affected by the mortality reductionis only the tip of the iceberg.

The decline in mortality associated with an air quality improvement is however only the tip of the iceberg. The well-known ‘pyramid of health effects associated with air pollution’ makes clear that the number of people affected by the most extreme effects (like the mortality reduction we have shown) is relatively small compared to those affected by non-fatal outcomes associated with air quality improvement: reduction of hospital admissions, doctor visits, restricted activity, medication use… This pyramid has been confirmed in many air pollution health impact assessments [46] (Fig 5) and amplifies the beneficial effects of a structural project like ‘Ringland’; it underlines the importance in terms of public health.

In this study we have focused on mortality in the general population and lung function of school age children. So low birth weight (LBW) and infant mortality were not taken into account. Although the effects are known to be relatively small, international evidence of both perinatal and lifelong effects of LBW on health, suggest that these effects could also be of major public health importance [47].

The improved lung function development we found in children because of decreased NO_2_ is clinically relevant. Impaired lung function in children has been associated with an increased risk of asthma [48]. Furthermore, a better lung function in early adulthood may decrease the risk of respiratory conditions, but the greatest benefits of improvements in lung function may occur later in life: a lung function lower than the predicted value for a healthy adult has been found to be associated with an increased risk of cardiovascular disease and an increased mortality rate [49]. Short-term exposure to PM_2.5_ and PM_10_ has also been associated with asthma exacerbations in adults and children, especially in children with allergic sensitization. Long-term exposure to air pollutants is associated with poorly controlled asthma [50].

A conceptual framework describes the most vulnerable segment of the population as being at greater risk of air-pollution-related mortality. For example, in a large community-dwelling cohort of older adults, strong evidence was found that of cumulative exposure to ozone or PM_10_ (of which 70% is PM_2.5_) was associated with decreased lung function [39]. The GERIE study showed that elderly people living in nursing homes with poor indoor air quality (coming partly from outsie), have an impaired respiratory health status. Elevated PM_10_ and NO_2_ were proven to influence the risk of coughing or general breathlessness [51]. Although the amplified health impact of air pollution in elderly people is hard to quantify, these studies suggest that the high number of elderly people living in nursing homes close to the Antwerp Ring Road (Table 6), could benefit even more from the predicted air quality improvement generated by the ‘Ringland project’.

**Table 6:** Nursing homes and their residents in the 1500 meter radius around the ring road.

Number of nursing homes	Number of elderly people
52	5,810

The calculated health impact of the PM_2.5_ improvement was slightly higher in the ‘Ringland South’ area than in the ‘Ringland North’ area (Fig 3 & Table 3). However, people living in the immediate vicinity of the ring road tend to have a lower socioeconomic profile, especially in the ‘Ringland North’ area (e.g. district ‘Borgerhout’), where the population densities can reach 11,000 inh/km² or more. These observations suggest that the health impact of the ‘Ringland project’ in these areas could be much higher, because people from lower socio-economic classes have a more compromised health status, and are therefore more affected by air pollution [52].

The annual mortality at the tunnel mouth areas is increased. The number of people negatively affected in these areas is 15,366, a relatively small number when compared to the 352,000 people living within a 1,500 meter radius of the ring road (Table 4). Although the overall positive effect on health remains—since a considerably larger number of people is exposed to improved air quality–, this tunnel mouth problem is not acceptable from a public health perspective. The current tunnel mouths of the ‘Ringland project’ should be modulated or moved further away from densely populated areas before the project can be implemented.

### Limitations

The ‘Ringland project’ air quality analysis used the well-established and internationally validated RIO-IFDM-street canyon model, taking into account changes in the traffic situation by 2020 and a prognosis of differences in the future vehicle fleet. However it remains only a prediction of PM_2.5_ and NO_2_ changes.

Furthermore the ‘Oosterweel Noord’ scenario was used as an example highway trajectory for both the ‘filtered tunneled ring road’ scenario as the ‘open air ring road’ scenario. One could argue that this is only one of the possible highway trajectories. However, for our HIA, different trajectories would probably not make a difference in trends, since our study focused on differences in air pollution next to the ring road close to the city center where the population density is highest. Differences in PM_2.5_ and NO_2_ were also most prominent next to the ring road in the center. Even in combination with other trajectories, within the ‘Ringland’ scenario, the entire urban ring road would still be covered, so the population exposed to decreased air pollution values would probably be more or less the same.

One of the largest sources of uncertainty concerns the evaluation of evidentiary support for causal interpretation of the current body of epidemiological work. Furthermore there is uncertainty introduced by the need to ‘translate’ results from one setting to another, e.g. USA to Europe, but also from national air quality changes to local changes [53].

Heterogeneity in PM_2.5_ effect estimates was found across studies, likely related to differences in particle composition, infiltration of particles indoors, population characteristics and methodological differences in exposure assessment and confounder control [54]. However, it was recently demonstrated that HIAs of traffic-related pollutants based upon PM_2,5_ seriously underestimate the health risks compared to an assessment based upon elemental carbon [55]. Furthermore six European experts recently concluded that ACS and SCS probably underestimate mortality caused by PM_2.5_ exposure [53]. This suggests that the health impact we predicted by the changes in PM_2.5_ will be more likely underestimated than overestimated.

There are still considerable knowledge gaps in the current literature on the health impact of PM_2.5_ and NO_2_ air quality improvement. This requires further research which should focus on expanding air pollution modeling to other vehicular pollutants, especially NO_x_, black carbon and PM_0.01_, therefore enhancing our ability to estimate the benefits of improved air quality. Recent evidence suggests that ultrafine particles (PM_0.01_)—of which 50% could be related to transport—are more ‘dangerous’ than the PM_10_ and PM_2.5_ fractions. These very small particles are shown to penetrate to the bloodstream of humans and induce atherosclerosis in test animals [56]. Unfortunately far more evidence is needed before this parameter can be used for a health impact assessment.

The health impact of environmental noise is a growing concern, but was not taken into account in this study. At least one million healthy life years are lost every year from traffic-related noise in Western Europe. The effects of noise pollution are insidious but debilitating: cognitive impairment in children, sleep disturbance, anxiety, but also hypertension and ischemic heart disease [57]. Therefore the global cardiovascular health impact of the ‘Ringland project’ is probably underestimated in our study. A higher health impact of the project could be expected if noise pollution is also taken into account.

Also not taken into account in our study is the health impact of the green space that the ‘Ringland project’ could provide in the newly created space on top of the covered ring road. The prevalence of cardiovascular risk factors and diabetes mellitus has been shown to be significantly lower among park users than among non-users in the Kaunas cohort study. An increased risk of non-fatal and fatal cardiovascular diseases (CVD) combined was observed for those who lived ≥629m from green spaces (hazard ratio (HR) = 1.36), and the risk of non-fatal CVD-for those who lived ≥347m and were not park users (HR = 1.66) [58].

A final limitation of our study is the incorporation of the health impact of the increased exposure to air pollutants for tunnel users. The concentration of air pollutants in tunnels is very high, compared to the concentrations outside, especially during daytime and rush hours [59]. On the other hand, the exposure time to the high levels of air pollutants in the tunnel is limited for the car users, compared to lifelong exposure to reduced concentrations for people living in the proximity of an open air highway. In a recent publication Swedish researchers present the prediction of the health impact for a planned (non-filtered) tunnel in Stockholm. They expect a pronounced negative health outcomes for people using the tunnel of 18 km on a daily basis [60]. A negative health impact for people using the ‘Ringland tunnel’ on a regular basis cannot be excluded and was not considered by our study. The negative health effects of short-term exposure to air pollution are well recognized, however several epidemiological data suggest that long-term exposure may be more important in terms of overall public health impact [8, 9].

## Conclusion

The expected change of PM_2.5_ by moving traffic from the entire Antwerp urban ring road into a tunnel—as proposed by the ‘Ringland project’–is associated with a considerable annual mortality decrease and an increase in life expectancy. Furthermore, the decrease of annual NO_2_ concentrations expected by the same ‘filtered tunneled ring road’ plan is associated with an improved lung function development in school age children, which is associated to long-term respiratory and other health benefits.

Although the overall positive effect remains, the tunnel mouths of the ‘Ringland project’ should be modulated or moved further away from densely populated areas, because of the expected increased mortality in these areas.

The cardiovascular health impact of the ‘Ringland project’ will probably be more pronounced than estimated in our study, if noise pollution and the positive effect of green space are also taken into account.

## Supporting Information

S1 File Supplementary Modeling Information. (DOCX)

S2 File Ringland Figure Permission 1a. (PDF)

S3 File Ringland Figure Permission 1b. (PDF)

S4 File Calculation Ringland Mortality Reduction and Life Expectancy. (XLSX)

S5 File Calculation Lung Function Development Children at Schools. (XLSX)

S6 File Calculation Cardiovascular and Lung Cancer Outcomes. (XLSX)

S7 File Addendum Scope Review. (DOCX)

## Abbreviations

ACS: American Cancer Society Cancer prevention study
CAFE: Clean Air for Europe study
IFDM: Immission Frequency Distribution Model
LBW: low birth weight
HIA: Health Impact Assessment
FVC: Forced Vital Capacity
HR: Hazard Ratio
RL-F: Ringland project with filtration
Ref 3-0-0: the reference scenario (Oosterweel Noord Highway without tunnel)
RR: Relative risks
SCS: Harvard Six Cities Study
TSP: Total Suspended Particles
