# Supplementary material for: Health Impact Assessment of a Predicted Air Quality Change by Moving Traffic from an Urban Ring Road into a Tunnel. The Case of Antwerp, Belgium
Source: PLoS One. 2016 May 11;11(5):e0154052. doi: 10.1371/journal.pone.0154052 (PMC4863966; doi:10.1371/journal.pone.0154052)

Supplementary modeling information:

**Health Impact Assessment of a Predicted Air Quality Change by Moving Traffic from an Urban Ring Road into a Tunnel. The Case of Antwerp, Belgium.**

Daan Van Brusselen^1^, Wouter Arrazola de Oñate^2^, Bino Maiheu^3^, Stijn Vranckx^3^, Wouter Lefebvre^3^, Stijn Janssen^3^, Tim S Nawrot^4,5^, Ben Nemery^5^, Dirk Avonts^1^.

1. The tunnel emission model

Here we provide some more details on the way in which the emissions from the tunnel complex are re-distributed to the tunnel portals. Due to conservation of mass, whatever is emitted inside the tunnels must also somehow leave the tunnel. We neglect wall deposition here as we assume this fraction is negliable w.r.t. the total emissions from the tunnel portal and would fall within the uncertainty of the dispersion modelling anyway. Given the complexity of the tunnel system, a full tunnel model was used. The tunnel was represented by a network of nodes and segments.


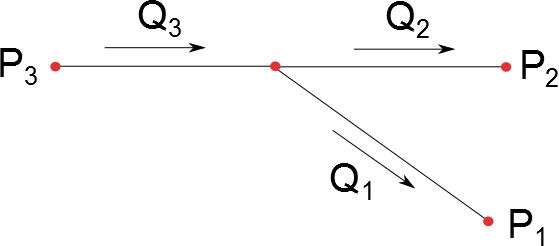

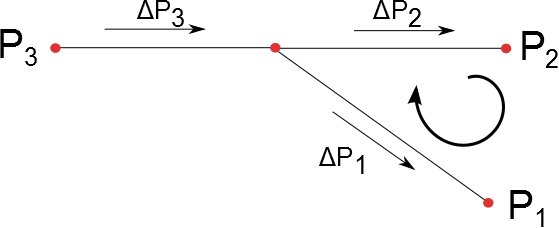


At each tunnel node conservation of mass was imposed : , where Q is the flow rate in the tunnel segment. On each segment, conservation of energy is imposed, where the pressure difference between two nodes equals the pressure loss/gain of the segment. As pressure losses for each of the segments, the wall friction is taken into account according to :

Where :

- : density of air ( 1.2 kg/m^3^ )
- : tunnel flowspeed [m/s]
- : resp. length of segment & tunnel hydraulic diameter ( , where A the frontale tunnelsurface and P the tunel perimeter ).
- : a dimension less friction constant, typically 0.015 – 0.025 for tunnels (Bring et al., 1997), which can be computed based upon the well known Moody-chart.

The pressure gain due to traffic is taken into account vai het traffic piston equation :

Where :

- : the frontale tunnelcross section
- : the traffic velocity in the tunnel
- : the flow speed in the tunnel
- : the drag-coefficient for vehicule type k, typically 0.35 for person cars, and 0.96 for lorries.
- : the effective frontal surface of vehicule type k, typically 2 m^2^ voor person cars and 6-7 m^2^ for lorries.
- : the total number of vehicules of type k in the tunnel segment.

We distuinguish different aerodynamic properties of the vehicules in the tunnel model :

|  | **Person cards** | **Light freight** | **Heavy freight** |
| --- | --- | --- | --- |
| *Aerodynamic drag coefficeënt, C_d_* | 0.35 | 0.70 | 0.96 |
| *Aerodynamic surface, A_eff_ [m^2^]* | 2.0 | 4.0 | 6.0 |

Through the coupling of pressure and flowrate ad the nodes, the entire tunnel system can be solved via a nonlinear system of equations, which can be solved iteratatively in MATLAB using the “fsolve” routine. As a results we obtain the pressures at the nodes and the flowrates at each of the segments, including the segments leading to the tunnel portals. We are therefore able to compute what fraction of the flowrate exists the tunnel at each portal and what fraction of the flowrate continued throught the tunnel. This provides us a way to redistribute the traffic emissions in the tunnel to the different portals along the trajectory. This redistribution of tunnel traffic emisisons is given in the table below where at each of the portals the fraction of the total tunnel traffic emission is given.

| Tunnel portal complex | Fraction of total emisisons |
| --- | --- |
| Kennedytunnel West | 14.6% |
| Scheldekaaien | 3.6% |
| Antwerpen Zuid | 5.6% |
| A12 JDV Noord | 0.3% |
| A12 JDV1 Zuid | 5.1% |
| E19 Craeybeckx-Noord | 1.0% |
| E19 Craeybeckx-Zuid | 10.1% |
| Singel | 1.5% |
| Berchem Kerk | 2.9% |
| Berchem Station | 2.6% |
| Plantin Moretus | 7.1% |
| E313 Rivierenhof | 15.7% |
| Sportpaleis | 5.0% |
| Groenendaal | 3.9% |
| Antwerpen Noord | 21.0% |

1. High resolution air quality model results

To illustrate the obtained resolution of the air quality model chain, a NO_2_ concentration map is presented here, showing the significant gradients in concentrations close to major roads, building footprints are shown in grey.


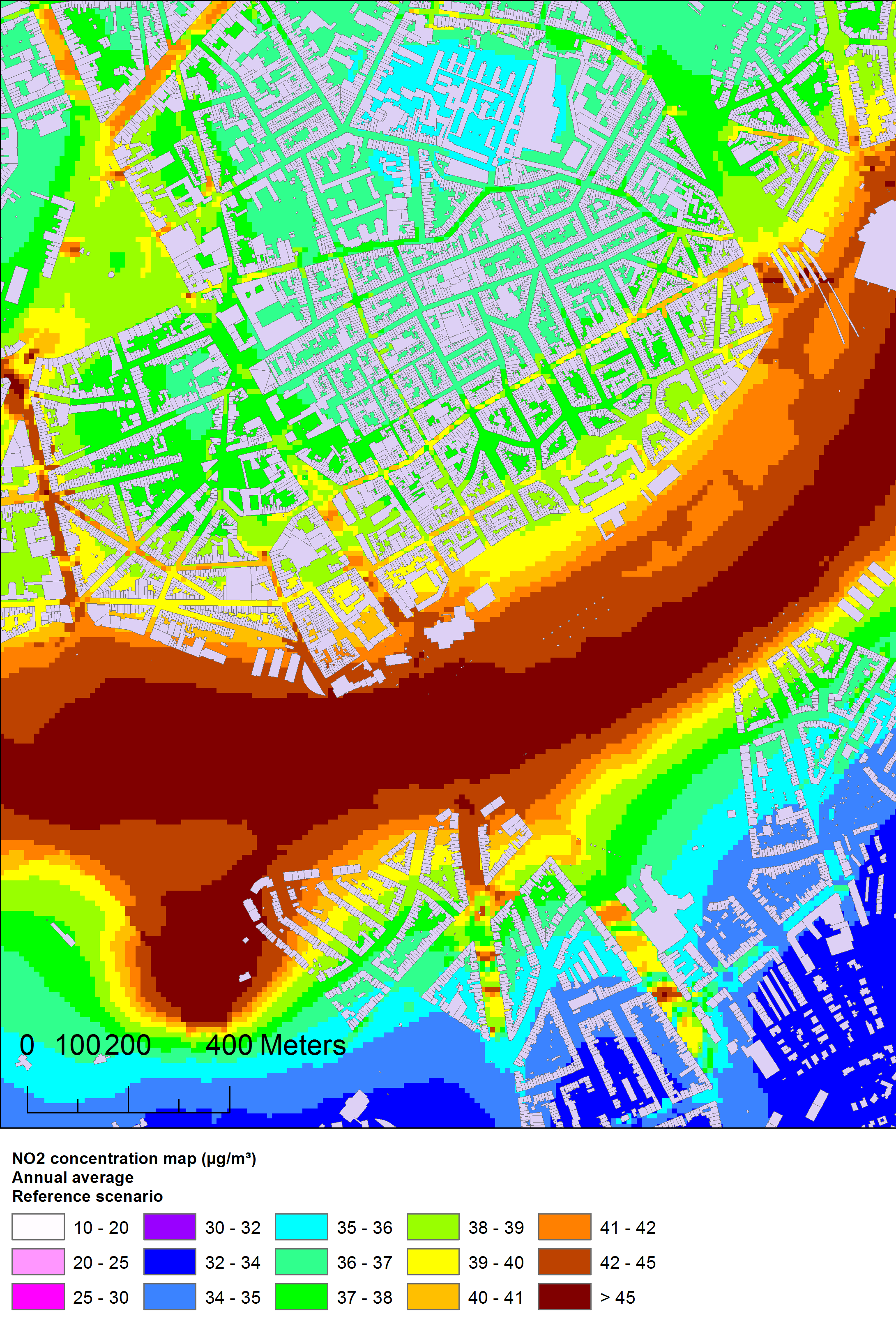

Supplement: S1 File — (DOCX) [file pone.0154052.s001.docx]
