# Supplementary material for: Health Impact Assessment of a Predicted Air Quality Change by Moving Traffic from an Urban Ring Road into a Tunnel. The Case of Antwerp, Belgium
Source: PLoS One. 2016 May 11;11(5):e0154052. doi: 10.1371/journal.pone.0154052 (PMC4863966; doi:10.1371/journal.pone.0154052)
Supplement: S3 File — (PDF) [file pone.0154052.s003.pdf]

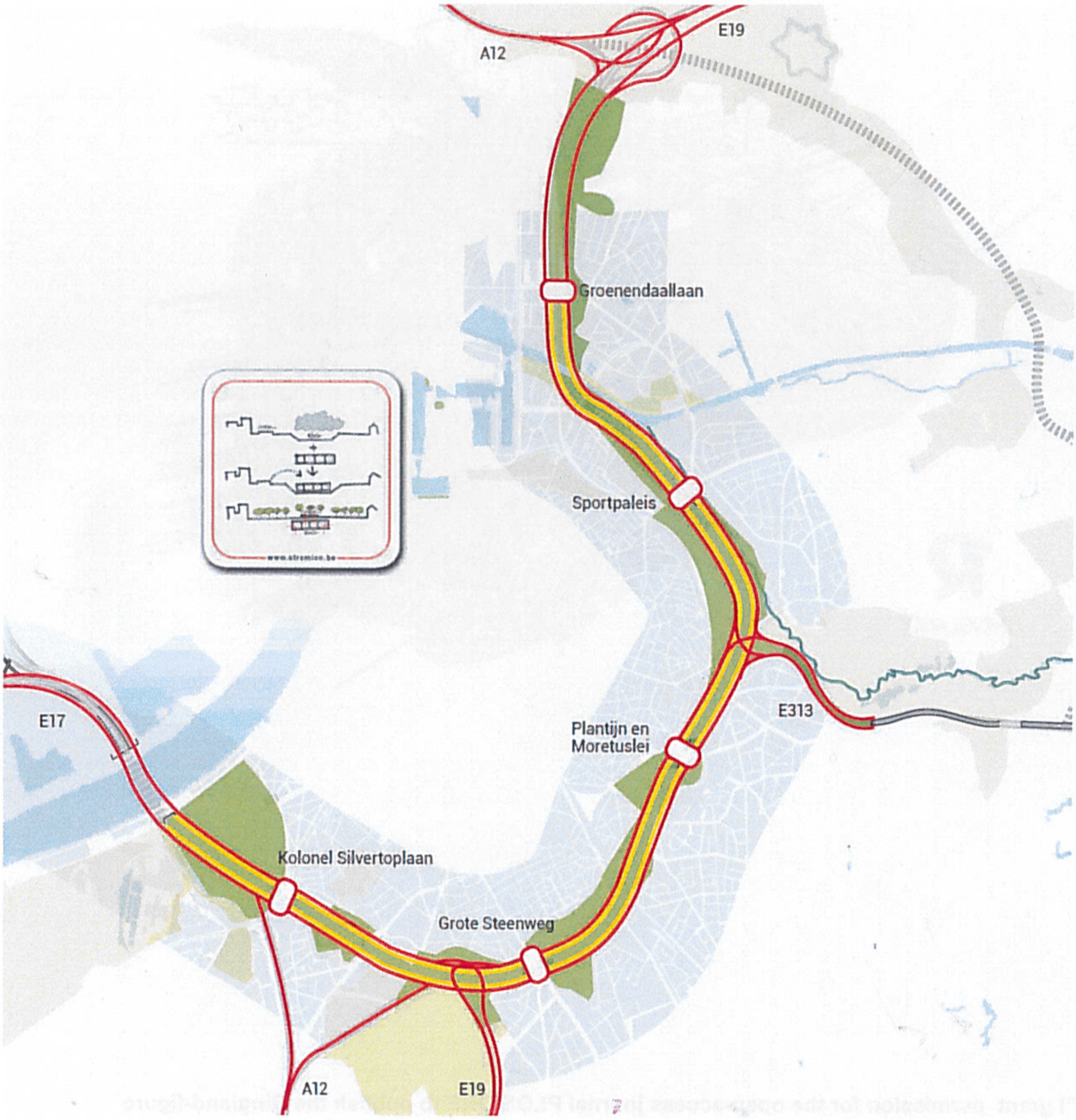

"I grant permission for the open-access journal PLOS ONE to publish the Ringland-figure (attached) under the Creative Commons Attribution License (CCAL) CC BY 4.0 (http://creativecommons.org/licenses/by/4.0/). I am aware that this license allows unrestricted use and distribution, even commercially, by third parties. I explicitely permit to publish the Ringland-figure (see attached) under a CC BY license."

Signature:

Peter Vermeulen, Het Ring Genootschap VZW (Ringland)
