# Supplementary material for: Health Impact Assessment of a Predicted Air Quality Change by Moving Traffic from an Urban Ring Road into a Tunnel. The Case of Antwerp, Belgium
Source: PLoS One. 2016 May 11;11(5):e0154052. doi: 10.1371/journal.pone.0154052 (PMC4863966; doi:10.1371/journal.pone.0154052)
Supplement: S7 File — (DOCX) [file pone.0154052.s007.docx]

**ADDENDUM 1**

Using the selection criteria described we identified 32 articles on health outcomes of PM_2,5_ and NO_2_ reduction effects that would be appropriate for evaluation in our study.

The scientific literature that assesses associations between air pollution and premature mortality and morbidity has rapidly expanded over the past decade, with several large-scale multi-city studies that extend or reanalyse earlier studies. We start with a description of the epidemiologic studies on PM_2,5_ and NO_2_ we evaluated, since this is the oldest and largest body of literature, continuing with studies on health improvement.

***a) EPIDEMIOLOGIC STUDIES***

Most notably, two large prospective cohort studies based in the United States, the American Cancer Society (ACS) Cancer Prevention Study, including an extended follow-up study, and the Harvard Six Cities Study (SCS) showed robust and statistically positive associations between long term exposure to concentrations of ambient air pollution and mortality from cardiopulmonary diseases after adjusting for smoking and other risk factors [1-4].

The Environment Protection Agency (EPA) has conducted benefit assessments for PM_2,5_ reduction and these assessments have also undergone peer-review of the analytical approaches used, including the choice of Concentration Response Function. The conclusion is generally that ACS and SCS remain the preferred basis to estimate mortality [5, 6]. At the same time it is important to note that European expert opinion regarding mortality and PM2,5 concluded that ACS and SCS probably even underestimate mortality [7].

The ACS is a prospective mortality study in 500.000 adults with a 16 year follow-up period. In this study each 10μg/m^3^ elevation in long-term average PM2,5 ambient concentrations was associated with approximately a 6%, 9% and 14% increased risk of all-cause, cardiopulmonary, and lung cancer mortality, respectively. The adjusted RR was 1,06 (95% CI 1,02-1,11) for all-cause mortality, 1,09 (95% CI 1,03-1,16) for cardiopulmonary mortality and 1,14 (95% CI 1,04-1,23) for lung cancer mortality. [1] In the Harvard Six Cities Study (SCS) an increased RR of 1,16 (95%CI of 1,07-1,26) in overall mortality associated with each 10μg/m^3^ increase in PM2,5 was found. PM2,5 exposure was also associated with lung cancer deaths (RR 1,27; 95% CI 0,96-1,69) and cardiovascular deaths (RR 1,28; 95%CI 1,13-1,44) [4].

A systematic review of the association between long-term exposure to ambient pollution and chronic diseases concluded that long-term exposure to PM2,5 increases the risk of cardiovascular mortality by approximately 12-14% per 10μg/m^3^ increase in PM_2,5_, independent of age, sex and geographic region [8]. A Canadian national level cohort-study found Hazard Ratios (HRs) for risk of non-accidental mortality and cardiovascular mortality associated with long-term exposure to PM_2,5_ similar to those reported in the ACS study: a HR of 1.15 (95%CI 1,13 – 1,16) for non-accidental mortality and a HR of 1.31 (95%CI 1,27 – 1,35) from ischemic heart disease [9]. There is a significant number of new studies on long-term air pollution exposure, covering a wider geographic area, including Asia. These recent study support associations found in previous cohort studies on PM_2,5_. A recent review, including these studies, found a pooled estimate expressed as excess risk per 10μg/m^3^ increase in PM_2,5_ exposure was 6% (95% CI 4-8%) for all-cause and 11% (95% CI 5-16%) for cardiovascular mortality. Long-term exposure to PM_2,5_ was more associated with mortality from cardiovascular disease (particularly ischemic heart disease) than from non-malignant respiratory diseases (pooled estimate 3% (95% CI 6-13%) [10].

In The Lancet, Beelen and colleagues recently presented a timely analysis of pooled data from 22 longitudinal cohort studies across Europe, including more than 360.000 people followed up for an average of 13,9 years (the ESCAPE study). The authors showed a 7% increase in natural cause mortality with each 5μg/m^3^ increase in PM_2,5_ concentration (HR 1,07; CI 1,02 - 1,13). HRs remained significantly raised even when only participants exposed to pollutant concentrations lower than the European annual mean limit value of 25μg/m^3^ or even below 20μg/m^3^ [11]. The same authors reported an 18% increase in lung cancer incidence for each 5μg/m^3^ increase in PM2,5 concentration in this cohort [12]. Given the strong relationship between cigarette smoking and lung cancer risk, evidence of an association between PM2,5 and lung cancer is more convincing when observed among never-smokers, compared with current or former smokers. A 26 year prospective follow-up study of 188.699 never smoking participants revealed that each 10μg/m^3^ increase in PM2,5 was associated with 15-27% increase in lung cancer mortality [13].

A Canadian study identified seven cohort studies (ACS; Six Cities Study; California Teachers Study; Nurses Health Study; Women’s Health Initiative; Adventist Study of Health and Smog and Netherlands Study of Cancer and Diet) that estimated the association between exposure to PM2,5 and mortality due to ischemic heart disease. The mean relative risk for a 10μg/m^3^ change in ambient PM2,5 concentration from these studies was 1.2 with a 90% CI of 0.98 – 1.48 [9]. A meta-analysis in 11 European cohorts from the ESCAPE project found that a 5μg/m^3^ increase in estimated annual mean PM2,5 was associated with a 13% increased risk of coronary events (HR 1,13; 95% CI 0,98 – 1,3). Positive associations were again detected below the current annual European limit of 25μg/m^3^. [14]

An analysis from 11 European cohorts within the ESCAPE project demonstrated that a 5-μg/m3 increase in annual PM2.5 exposure was associated with 19% increased risk of incident stroke (hazard ratio 1.19, 95% CI: 0.88, 1.62). The results were robust to adjustment for an extensive list of cardiovascular risk factors and noise co-exposure. The association with PM2.5 was apparent among those ≥ 60 years of age (HR = 1.40, 95% CI: 1.05, 1.87), among never-smokers (HR = 1.74, 95% CI: 1.06, 2.88), and among participants with PM2.5 exposure < 25 μg/m3 (HR = 1.33, 95% CI: 1.01, 1.77) [15]. In a Canadian national level cohort-study however, there was little to no association with cerebrovascular mortality [9].

**Vulnerable groups.**

According to the American National Ambient Air Quality Standards the PM2,5 related health effects were found to be stronger in susceptible population groups, such as the elderly, young children, and people with preexisting cardiovascular and respiratory conditions [16]. The groups most susceptible to health effects of air pollution are children, adults over 65 years of age, persons with chronic diseases such as asthma, chronic obstructive pulmonary disease, and cardiac ischemia, and pregnant women because of prenatal exposure of the fetus [17, 18].

The Committee on Environmental Health of the American Academy of Pediatrics issued a policy statement in 2004 emphasizing the link between ambient air pollution and children’s health. Children are known to be more vulnerable to the adverse health effects of air pollution due to their higher minute ventilation, immature immune system, involvement in vigorous activities, the longer periods of time they spend outdoors and the continuing development of their lungs during the early postnatal period [19]. Exposure to air pollutants during pregnancy and early stages of life is associated with premature birth, delayed intrauterine growth, low birth weight, early death syndrome and infant mortality [20]. A multi-country evaluation concluded that term low birth weight (LBW) was positively associated with a 10μg/m^3^ increase of PM2,5 (OR 1,1; 95% CI 1,03 -1,18) during the entire pregnancy [21].

Infant mortality remains a major contributor to childhood mortality worldwide, despite significant declines over the last two decades. Although more evidence is needed to confirm and clarify findings, a systematic review of the literature on the association between ambient air pollution and infant mortality, observed a consistent association between PM and postnatal mortality due to respiratory causes, as well as sudden infant death syndrome [22].

While exposures to urban PM_2,5_ and soot-black carbon (diesel exhaust) have been associated with asthma exacerbations, there is limited evidence on whether these pollutants are associated with the new development of asthma and allergy among young inner city children. Recently however, a positive association was found between PM_2,5_ and new wheeze in an urban prospective cohort study of children between 5 and 7 years old. An adjusted odds ratio of 1.51 (95% CI 1,05 – 2,16) per interquartile range was found for indoor PM_2,5_ concentrations, much of which penetrated readily from outdoor sources in this urban environment [23].

A prospective birth cohort study in Germany found strong positive associations between the distance to the nearest main road and asthmatic bronchitis, hay fever, eczema and allergic sensitization, with the highest odds ratio (OR) for children living less than 50 meters from busy roads. For PM_2,5_ absorbance, stastically significant effects were found for asthmatic bronchitis (OR 1,56; 95% CI 1,03-2,37), hay fever (OR 1,59; 95% CI 1,11-2,27) and allergic sensitization to pollen (OR 1,40; 95% CI 1,20-2,64). NO2 exposure was associated with eczema, whereas no association was found for allergic sensitization (OR 1,18; 95% CI 1,00-1,39) [24].

A prospective study in Southern California demonstrated that exposure to EC, PM_2,5_, acid vapors and NO_2_ was associated with subsequent deficits in lung function among children ages 10 to 18 years. Children who lived 500m of a freeway had substantial deficits in 8-year growth of forced expiratory volume in 1 second (FEV1, -81ml, 95% CI -143 to -18), compared with children who lived at least 1500 meter from a freeway. PM2,5 and NO2 were independenty associated with reduced lung function [25].

***b) STUDIES ON AIR QUALITY IMPROVEMENT***

Findings from the epidemiologic studies have supported the implementation of health protective policies among populations exposed to air pollution [26]. A variety of studies has now also documented the relationship between decreased exposure to air pollution (partly because of measures taken by governments) and decreases in population mortality and morbidity as well as increases in life expectancy [27, 28]. In an extended follow-up of the Harvard Six Cities Study by Laden et al, an improved overall mortality (RR 0,73; 95% CI 0,57-0,95) was associated with a decreased mean PM2,5 of 10μg/m^3.^ [4]. Pope and colleagues reported that PM2,5 concentrations fell by a third from the early 1980s to the late 1990s across major US metropolitan areas, with each 10μg/m^3^ reduction associated with an increase in life expectancy of 0.61+/-0.20 year (P=0.004) [28]. In a Japanese study the effect of a diesel emission control law on mortality rates in 23 wards of Tokyo metropolitan area was evaluated. Even after adjustment of longer-time trend, mortality rate from cerebrovascular disease was reduced by 8,5% (p <0.001) while PM2,5 concentration was reduced by 2,5μg/m^3^ over a 5 year period of time. However, the declines in other cause-specific mortality became equivocal [29].

Since air-pollution has been trending downward over the last several decades in California – as a result of implementation of air-quality policies – improvements in 4-year growth of both FEV1 and FVC could be assessed by the Gauderman Follow-Up Study. They were both most strongly associated with declining levels of NO2 and PM2,5. These associations persisted after adjustment for several potential confounders. A decrease of 14,1 ppb in NO2 was associated with estimated mean FEV1 growth of 91,4ml (95% CI 47,9 – 134,9). A decrease of 12,6μg/m^3^ in PM2,5 was associated with an estimated mean FEV1 growth of 65,5ml (95% CI 17,1 – 113,8) [30].

**Concentration response curve**

Taking into account the higher described inclusion criteria, we primarily selected studies on air quality improvement for ‘translation’ to the Antwerp situation. We identified the *extended follow-up of the Harvard Six Cities Study by Laden et al.* for the calculation of the changes in mortality, the study of *Pope at al.* as the most reliable study for the calculation of life expectancy, and the *Gauderman et al.* study as the most reliable to predict changes in childhood lung function [4, 28, 30].

The Concentration Response Functions used in our study correspond to the Relative Risks (RR) – or a pooled RR – found in prospective air quality improvement studies that show a relevant health gain associated with a decrease in PM_2,5_ and NO_2_ outdoors.

However, since the scarcity of studies on air quality improvement, we also used the

RR, found in observational epidemiologic studies assessing health effects and

changes in air pollution. Therefor we have used 2 meta-analyses of

epidemiologic studies about air pollution to assess the differences in lung cancer

mortality and the incidence of myocardial infarctions. [31, 32]

1. Pope CA BR, Thun MJ, Calle EE, Krewski D, Ito K, Thurston GD. Lung cancer, cardiopulmonary mortality, and long-term exposure to fine particulate air pollution. JAMA, 2002; 287:1132–1141.

2. Krewski D JM, Burnett RT, Ma R, Hughes E, Shi Y et al. Extended follow-up and spacial analysis of the American Cancer Society Study linking particulate air pollution and mortality. Res Rep Health Eff Inst 2009; 140:5-136

3. Dockery DW PCI, Xu X, Spengler JD, Ware JH, Fay ME et al. An association between air pollution and mortality in 6 US cities. N Eng J Med 1993; 329:1753-1759.

4. Laden F SJ, Speizer FE, Dockery DW. Reduction in fine particulate air pollution and mortality: extended follow-up of the Harvard Six Cities Study. Am J Respir Crit Care Med 2006; 173(6): 667-672.

5. Hall JV BV, Lurmann FW. Measuring the gains from improved air quality in the San Joaquin Valley. Journal of Environmental Management 2008; 88:1003-1015.

6. Kaiser J. Air pollution. Panel backs EPA and ‘six cities’ study. Science 2000; 289(5480): 711.

7. Cooke RM WA, Tuomisto JT, Morales O, Tainio M, Evans JS. A probabilistic cheracterization between fine particulate matter and mortality: elicitation of European experts. Environ Sci Technol 2007; 41:6598-6605.

8. Chen H GM, Villeneuve PJ. A systematic review of the relation between long-term exposure to ambient air pollution and chronic diseases. Rev Environ Health 2008; 23(4):243-297.

9. Crouse DL PP, van Donkelaar A, Golberg MS, Villeneuve PJ, Brion O et al. Risk of conaccidental and cardiovascular mortality in relation to long-term exposure to low concentrations of fine particulate matter: a Canadian national-level cohort study. Environ Health Perspect 2012; 120(5):708-714.

10. Hoek G RM, Beelen R, Peters A, Ostro B, Brunekreef B, Kaufman JD. Long-term air pollution exposure and cardiorespiratory mortality: a review. Environmental Health 2013; 12(43):1-15.

11. Beelen R R-NO, Stafoggia M, Andersen ZJ, Weinmayr G, Hoffmann B et al. Effects of long-term exposure to air pollution on natural-cause mortality: an analysis of 22 European cohorts within the multicentre ESCAPE project. Lancet 2013; 383:785-795.

12. Raaschou-Nielsen O AZ, Beelen R, Samoli E, Stafoggia M, Weinmayr G et al. Air pollution and lung cancer incidence in 17 European cohorts: prospective analyses from the European Study of Cohorts for Air Pollution Effects (ESCAPE). Lancet Oncol 2013; 14:813-822.

13. Turner MC KD, Pope III CA, Chen Y, Gapstur SM, Thun MJ. Long-term ambient fine particulate matter air pollution and lung cancer in a large cohort of never-smokers. Am J Resp Crit Care Med 2011; 184: 1374-1381.

14. Cesaroni G1 FF, Stafoggia M, Andersen ZJ, Badaloni C, Beelen R et al. Long term exposure to ambient air pollution and incidence of acute coronary events: prospective cohort study and meta-analysis in 11 European cohorts from the ESCAPE Project. BMJ 2014; 21; 348:f7412.

15. Stafoggia M1 CG, Peters A, Andersen ZJ, Badaloni C, Beelen R et al. Long-term exposure to ambient air pollution and incidence of cerebrovascular events: results from 11 European cohorts within the ESCAPE project. Environ Health Perspect 2014 Sep;122(9):919-25.

16. Johnson PR, Graham JJ. Fine particulate matter National Air Quality Standards: public health impact on populations in the Northeatern United States. Environ Health Perspect 2005; 113:1140-1147.

17. Brunekreef B, Holgate ST. Air pollution and health. Lancet 2002; 360:1233-1242.

18. Kampa M, Castanas E. Human health effects of air pollution. Environ Pollut 2008; 151:362-367.

19. Buka I KS, Orornio-Vargas AR. The effects of air pollution on the health of children. Paediatr Child Health 2006; 11(8):513-516.

20. Lacasaña M EA, Ballester F. Exposure to ambient air pollution and prenatal and early childhood health effects. J Epidemiol 2005; 20: 183-199.

21. Dadvand P PJ, Bell ML, Bonzini M, Brauer M, Darrow LA et al. Maternal exposure to particulate air pollution and term birth weight: a multi-country evaluation of effect and heterogeneity. Environ Health Perspect 2013; 121:367-373.

22. Glinianaia SV RJ, Bell R, Pless-Mulloli T, Howel D. Does particulate air pollution contribute to infant death? A systematic review. Environ Health Perspect 2004; 112: 1365-71.

23. Jung KH HS, Yan B, Moors K, Chillrud N, Ross J et al. Childhood exposure to fine particulate matter and black carbon and the development of new wheeze between ages 5 and 7 in an urban prospective cohort. Environ Int 2012; 45:44-50.

24. Morgenstern V ZA, Cyrys J, Brockow I, Koletzko S, Krämer U et al. Atopic diseases, allergic sensitization and exposure to traffic-related air pollution in children. Am J Respir Crit Care Med 2008; 177-1-7.

25. Gauderman WJ A, Gilliland F, Vora H, Thomas D, Berhane K et al. The effect of air pollution on lung development from 10 to 18 years age. N Eng J Med 2004; 351:1057-67.

26. WHO. Air quality guidelines global update 2005. Germany: World Health Organization, 2006.

27. Leksell I, Rabl A. Air pollution and mortality: quantification and valuation of years of life lost. Risk Anal 2001; 21:843-857

28. Pope CA 3rd EM, Dockery DW. Fine-particulate air pollution ad life expectancy in the United States. N Eng J Med 2009; 360: 376-386.

29. Yorifuji T KI, Kaneda M, Takao S, Kashima S, Doi H. Diesel vehicle emission and death rates in Tokyo, Japan: a natural experiment. Science of the total environment 2011; 409: 3620-3627.

30. Gauderman WJ UR, Avol E, Berhane K, McConnell R, Rappaport E et al. Association of improved air quality with lung development in children. N Engl J Med 2015; 372(10):905-913.

31. Hamra GB GN, Cohen A, Laden F, Raaschou-Nielsen O, Samet JM, Vineis P, Forastiere F, Saldiva P, Yorifuji T, Loomis D. Outdoor particulate matter and lung cancer: a systematic review and meta-analysis. Environ Health Perspect 2014; 122(9): 906-11.

32. Nawrot T. Meta-analysis of PM2,5 impact on myocardial infarctions and acute coronary syndrome. In: Milieubaten of milieuschadekosten, waarderingsstudies in Vlaanderen. LNE, Department of environment, nature and energy. Flanders; 2007.
